# The effect of an image of watchful eyes on the evaluation of the appearance of food

**DOI:** 10.7717/peerj.9804

**Published:** 2020-08-28

**Authors:** Kenichi Shibuya, Mana Miyamoto, Risa Santa, Chihiro Homma, Sumire Hosono, Naoto Sato

**Affiliations:** 1 Department of Health and Nutrition, Niigata University of Health and Welfare, Niigata, Japan; 2 Department of Health and Nutrition, Yamagata Prefectural Yonezawa University of Nutrition Sciences, Yonezawa, Yamagata, Japan

**Keywords:** Food appearance, Taste, Computer-based-evaluation, Eye-image

## Abstract

It is known that an eye-like image promotes generosity. It is also known that the evaluation of the visual deliciousness of food is improved in the presence of an emotionally positive stimulus. The purpose of the present study was to examine whether the presence of open eyes (OPEN) causes generous behavior altering the evaluation of the visual deliciousness of food, and how the images of open and closed eyes (CLOSED) affect human emotions. Seventeen women participated in the present study. A picture of food was presented on a computer screen, and the participants predicted and evaluated its visual deliciousness. An image of OPEN or that of CLOSED was presented simultaneously with a picture of food. There was a significant difference between the OPEN and CLOSED conditions, as demonstrated by the scores on a nine-point Likert scale for visual deliciousness; the ratings in the OPEN condition were significantly higher than those in the CLOSED condition (*p* = 0.004). There was no significant difference in the image of watchful eyes for the perceived relaxation state; the ratings in the OPEN condition were not significantly higher than those in the CLOSED condition (*p* = 0.716). The results of the present study revealed that the evaluation of the visual deliciousness of food based on its appearance was likely due to the presence of an image of open watchful eyes, increasing the perceived visual deliciousness of the food without any changes in the participants’ emotions.

## Introduction

Humans behave differently in the presence of others. Previous studies have repeatedly examined whether individuals become generous when they are watched by eye-like images (Nettle et al., 2013; Sparks & Barclay, 2013). Kawamura & Kusumi (2017) demonstrated that compared to no eye-like images, eye-like images on computer screens encouraged people to donate higher amounts. In addition, various research groups have repeatedly verified that individuals become generous when they feel that they are being watched by eye-like images (Haley & Fessler, 2005; Nettle et al., 2013; Barclay & Willer, 2007). A possible reason for maintaining generosity is the motivation to maintain a reputation (Alexander, 1987; Roberts, 1998). It is known that people feel the urge to manage their reputation when they are the focus of others’ attention (e.g., when one is not being watched) as compared to when they are not (e.g., when the eyes are closed).

On the other hand, the evaluation of food tastes tends to be associated extrinsic factors such as typeface, packaging shapes, positive valance, speech sounds and music (Knöeferle & Spence, 2012; Pathak, Calvert & Motoki, 2020; Motoki et al., 2020; Reinoso-Carvalho et al., 2019; Mesz, Trevisan & Sigman, 2011; Ngo et al., 2013; Salgado-Montejo et al., 2015; Velasco et al., 2014, 2015). Wang & Spence (2017) proposed that emotionally positive stimuli improve taste evaluations, regardless of the sensory modalities of the stimuli.

At present, it is unclear whether people act in such manner during a food-taste evaluation, when they are shown an image of eyes. It is possible to examine whether the participants have developed generosity in response to the presence of the image of eyes if the participants are asked to rate the evaluation of visual deliciousness in the presence of pictures of open and closed eyes (CLOSED). We hypothesized that the presence of a picture of open eyes (OPEN) would cause generosity, even if there was no difference in emotional effects between OPEN and CLOSED. The purpose of the present study was to examine whether the presence of an image of OPEN caused generosity, by using pictures of food.

## Methods

### Participants

Seventeen females aged between 18 and 22 years (20.8 ± 1.4 years) participated in the present study. Consent was obtained from all participants before the experiment was conducted. This study was approved by the Ethics Committee of Niigata University of Health and Welfare (Approval No. 18046-180720). All participants were naïve to the protocol of this study. The contents of the research were explained verbally to all the participants. Written informed consent was obtained from each participant after a full explanation of the nature of the study procedure and its noninvasiveness.

### Procedure

Upon arrival at the laboratory, to create a resting state, the participants were asked to sit in front of a computer, in a small room, for over 15 min. They were assured that their responses would remain completely anonymous as ID numbers were being used to manage the data. Then, they were given instructions about the tasks being conducted in the experiment. The experimenter then left the room to ensure the participants’ anonymity during the experiment. The participants performed the tasks facing the computer. All tasks were carried out using a computer program written on PsychoPy (Peirce et al., 2019). During the task, the top half of the computer screen displayed an image of OPEN or CLOSED (indicating OPEN and CLOSED conditions respectively). In the bottom half of the computer screen, images of food were presented randomly. The participants needed to evaluate the taste based on the appearance of these food images and the perceived relaxation state as soon as possible, after the images of eyes and food had disappeared, using a nine-point Likert scale (Lavin & Lawless, 1998). Each participant conducted 180 trials: 30 foods × 3 times × 2 conditions. The answers were stored on the system and analyzed later. The stimuli were presented on a 17″ (inch) CRT monitor (LCS 172VXL; NEC, Japan) with a resolution of 1,024 × 768 pixels and a refresh rate of 100 Hz. The presentation of the stimuli and the collection of data were controlled using a computer (M8-D; NEC, Japan). The visual stimuli comprised a fixation point, command cursors for rating, and images of eyes and food. Stimuli were presented at a viewing distance of 40 cm. The luminance of the fixation point was 91.0 cd/m^2^. The command cursors were white boxes surrounding each rating value (0.95 × 1.89°; 91.0 cd/m^2^) and the selected box was filled in white color. We used colorful pictures (12.1-degree × 12.1-degree) of food. The visual stimuli included 180 experimental trials: 90 OPEN and 90 CLOSED. Each trial commenced with a central fixation cross shown for 1,000 ms, followed by eyes and food images for 2,000 ms. Each food image was presented four times, and each of the 30 food images appeared for the same amount of time. Immediately after the image pair disappeared, a cross appeared at the center of the screen. Participants were instructed to look at the fixation cross at the start of each trial and to evaluate the taste based on the appearance of these food images and the perceived relaxation state as soon as possible, after the images of eyes and food had disappeared pressing a button from 1 to 9 on the keyboard as quickly and accurately as possible immediately after the image pair disappeared. The order in which the images were presented was randomized for each participant.

### Statistical analysis

All data are shown as the mean ± SD. Statistical analysis was conducted using R version 3.5.0 and the lmerTest package (Kuznetsowa, Brockhoff & Christenen, 2017). A generalized linear mixed model (GLMM) was used to avoid type 1 errors due to data nesting and violation of sphericity (Maruyama et al., 2014). The images of eyes (OPEN and CLOSED) were set as fixed effects, while participant IDs and food images were set as random effects. The Satterthwaite approximation was used as the degree of freedom estimation method (Satterthwaite, 1946) in the analysis of variance (ANOVA) from the results of GLMM. After the ANOVAs of the images of eyes, we used the analysis of least squares means for the evaluation of the statistical differences between these two conditions. Statistical significance was accepted when *p*-values were <0.05.

## Results

**Figure 1:**
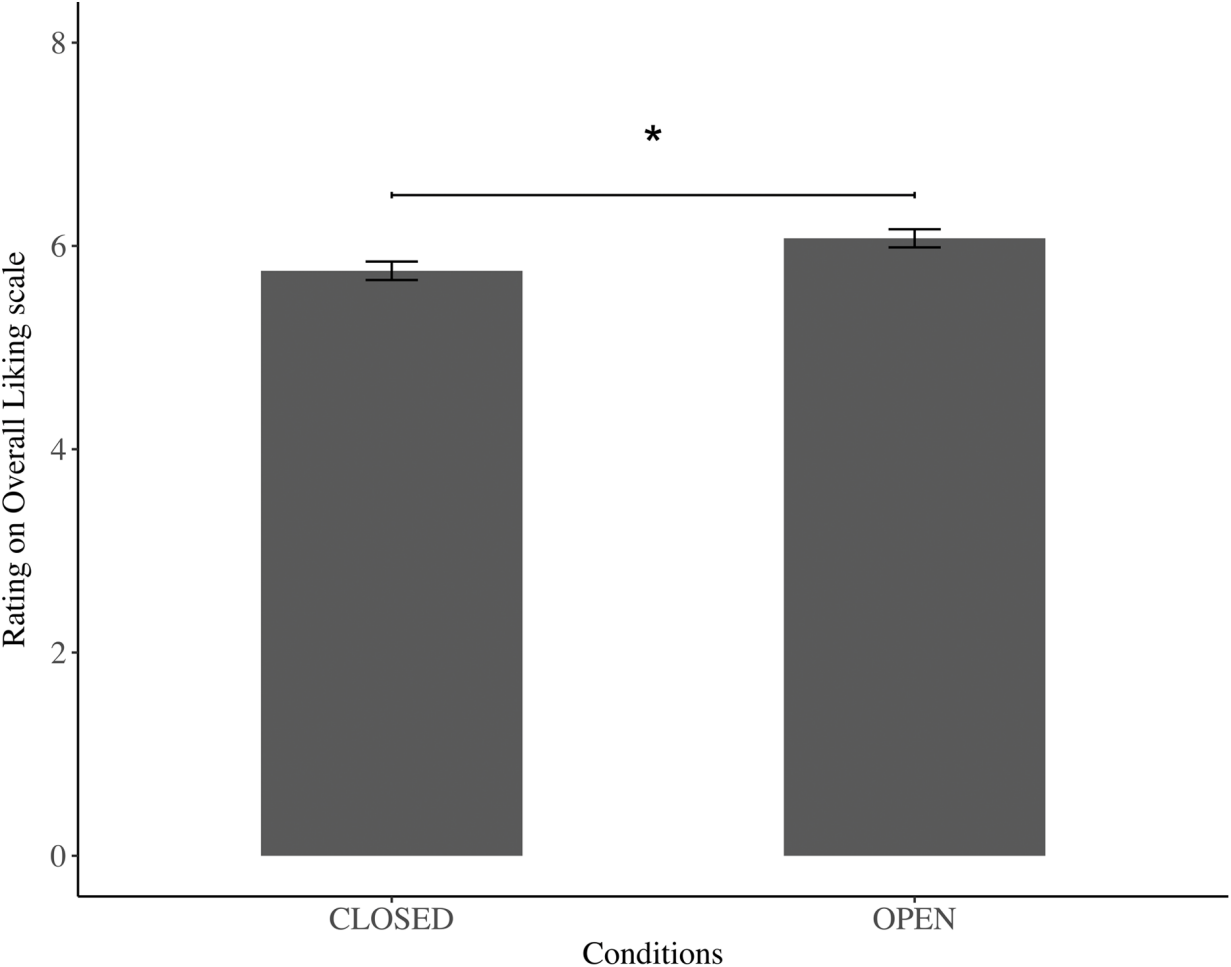
Evaluation of open-eye and closed-eye conditions. Values are means ± SE. The asterisk shows a significant difference between the OPEN and CLOSED conditions (*p* < 0.05).

Figure 1 shows the taste ratings on a nine-point Likert scale for the OPEN and CLOSED conditions. The average scores of the evaluation for food were 5.484 ± 2.481 in the CLOSED condition and 5.944 ± 2.454 in the OPEN condition.

There was a significant difference in the overall ratings of images of watchful eyes and CLOSED, on the nine-point Likert scale; the ratings in the OPEN condition were significantly higher than those in the CLOSED condition (*F* = 8.114, *p* = 0.005, 95% CIs [0.100–0.543]).

**Figure 2:**
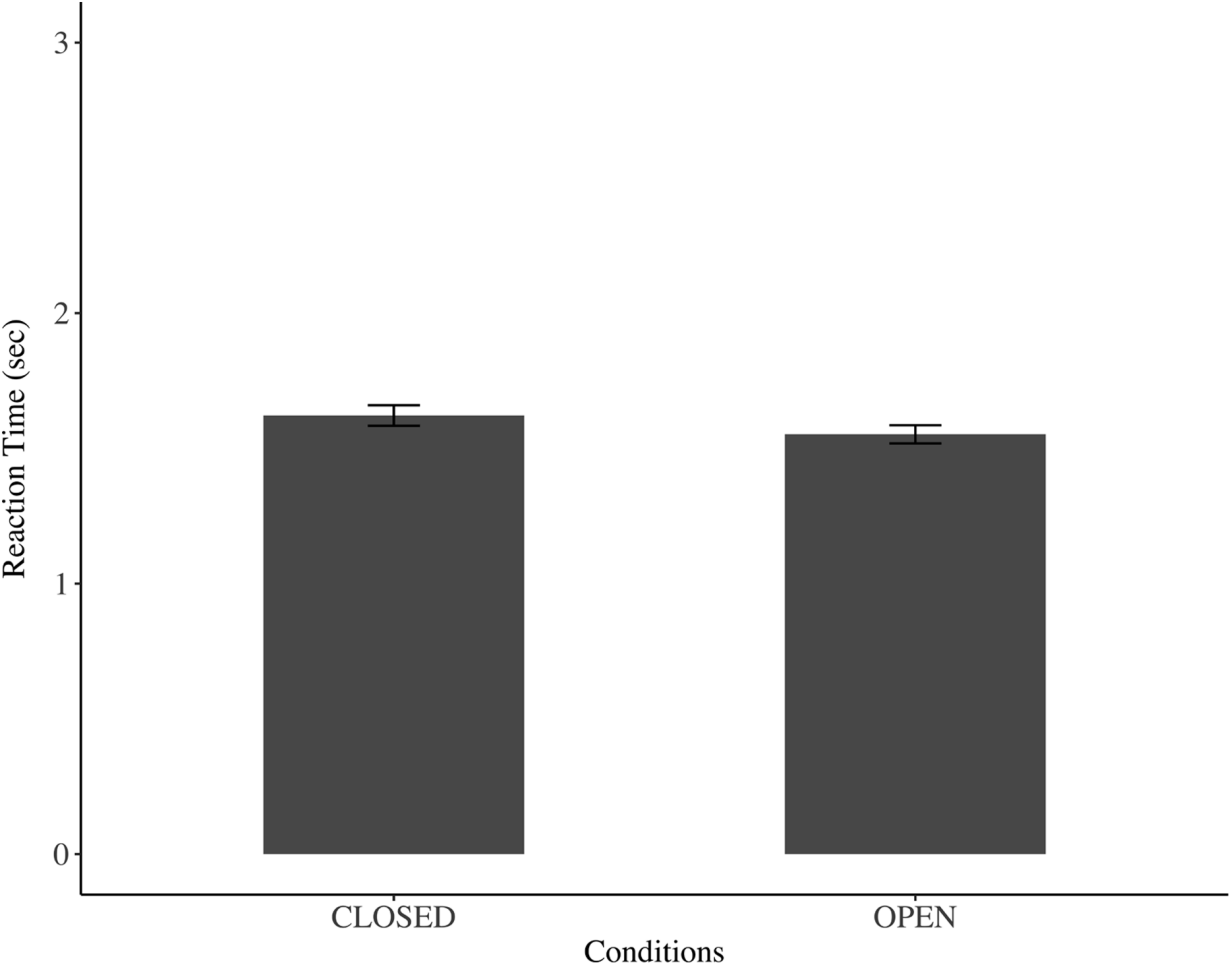
Mean response time in open-eye and closed-eye conditions. Values are means ± SE. There was no significant difference between the OPEN and CLOSED conditions (*p* > 0.05).

Figure 2 shows the reaction time for the OPEN (1.530 ± 0.008 s) and CLOSED conditions (1.601 ± 0.009 s). There was no significant difference in reaction time concerning the image of watchful eyes on reaction time (*F* = 2.477, *p* = 0.115, 95% CI [−0.017 to 0.158]). In addition, the average scores of the perceived relaxation state were 4.003 ± 1.137 in the CLOSED condition and 3.983 ± 0.970 in the OPEN condition. There was also no significant difference in the image of watchful eyes for the perceived relaxation state; the ratings in the OPEN condition were not significantly higher than those in the CLOSED condition (*F* = 0.132, *p* = 0.716, 95% CIs [−0.087 to 0.126]).

## Discussion

The purpose of the present study was to examine whether the presence of an image of OPEN causes generosity in evaluation of food tastes, by using pictures of food. The main finding of the present study was that the evaluation of the deliciousness of food based on its appearance was better in the presence of open-eyes images than in the presence of closed-eyes images, while there was no difference in perceived relaxation between those conditions. In other words, the results of the present study suggested a change in the generosity of participants in the presence of an image of OPEN, even though there was no emotional difference between the OPEN and CLOSED conditions. It is known that an image of OPEN creates generosity in the behavior of the participants, while emotional stimuli influence the evaluation of the taste of food presented on the computer screen.

Until now, the mood and relaxed state of mind have been cited as factors influencing the evaluation of food. (Baumeister & Leary, 1995; Hetherington et al., 2006; Hetherington, 2007). In addition, Wang & Spence (2017) proposed that emotional cues play an important role in evaluating the taste of the food and that taste ratings are higher when the cues are emotionally positive compared to the cues that are emotionally negative. Boothby, Clark & Bargh (2015) also demonstrated that the “ice-break effect” is necessary for inducing a positive taste perception. The results of the present study revealed that the evaluation of food taste was affected by the mere presence of an open-eyed image. An open-eyed image would not promote the “ice-break effect” any more than the image of CLOSED because there was no significant difference in the perceived relaxation between the OPEN and CLOSED conditions. Other previous studies also examined the effect of the image of OPEN by manipulating norm information (Bateson, Nettle & Roberts, 2006; Kawamura & Kusumi, 2017). Kawamura & Kusumi (2017) conducted an experiment to determine whether an image of OPEN increases the donation in a laboratory. The results of their study supported the “good reputation hypothesis”. In the real world, Bateson, Nettle & Roberts (2006) reported that an image of eyes motivates cooperative behavior because it induces a perception in participants of being watched. The results of the present study are consistent with those of previous studies (Bateson, Nettle & Roberts, 2006; Kawamura & Kusumi, 2017).

In the present study, we did not compare the evaluation scores for foods between the CLOSED condition and non-eye stimuli. In studies, conducted previously, using eye-like images, it was reported that there was no difference between the effects caused by the presence of CLOSED and non-eye stimuli (i.e., averted gaze eye, flowers, etc.) (Mansei, Van Lange & Pollet, 2016; Kawamura & Kusumi, 2017). These results suggested that closed-eyes stimuli did not significantly promote generous behavior than exposure to images such as flowers. For social influence to occur, the eyes need to be watching the participants and not being closed or looking away. In the present study, although we used an eye-like image, not actual human eyes, there was a significant social influence on the evaluation of visual deliciousness. However, it remains unclear whether the same results can be obtained when using actual human eyes. Further studies, in the future, are needed to clarify the effect of human eyes on food evaluations.

Various studies have been conducted to determine the effect of images of open watchful eyes; there are reports that people are more generous when they notice such images (Haley & Fessler, 2005; Oda et al., 2011). However, some studies have denied such effects (Matsugasaki, Tsukamoto & Ohtsubo, 2015; Raihani & Bshary, 2012; Tane & Takezawa, 2011). It has also been pointed out that cultural influences are involved in these behaviors (Raihani & Bshary, 2012). Gelfand et al. (2011) pointed out that people follow relatively rigid norms and have low tolerance toward behavior that deviates from these norms. Although the present study was conducted on a considerably small-scale, on one gender (female) only, it demonstrated that subconscious cues such as the images of eyes can enhance generosity. However, if there is a significant difference in the way an image of eyes is perceived by males and females, the results would be different. It is possible that in males too, the subtle cues may evoke the generosity, observed in this experiment, because the images of eyes induced a perception of being watched. Even if relaxation would influence food preference, the effect of relaxation might depend on food type as proposed by Biswas, Lund & Szocs (2019). And if the experimenters asked participants “positive arousal”, the results of the present study would be changed as suggested by Kaneko et al. (2019). The present study was conducted in the laboratory, not in the real world. As proposed by Spence, Mancini & Huisman (2019), the results in the laboratory using digital devices also in the present study would differ from the results in the real world. Further studies are needed to examine the results of the present study in the real world and with a large sample size.

In addition, the findings in the present study were based on the within-participants design. The participants might know the hypotheses behind the experiments. Future research should be based on the between-participants design.

## Conclusion

In conclusion, the main finding of the present study was that the evaluation of the deliciousness of food based on its appearance was better in the presence of an image of OPEN than of CLOSED. The results of the present study revealed that the evaluation of the visual deliciousness of food based on its appearance was likely due to the presence of an image of open watchful eyes, increasing the perceived visual deliciousness of the food. Overall, the results of the present study suggested that the image of OPEN motivated the participants to behave generously even if there was no difference in the emotional effects of the OPEN and the CLOSED.

## Supplemental Information

10.7717/peerj.9804/supp-1 Supplemental Information 1 Raw data.

## Additional Information and Declarations

### Data Availability

The following information was supplied regarding data availability: The raw data are available in the Supplemental File.
